# Comparison of novel visceral obesity indexes with traditional obesity measurements in predicting of metabolically unhealthy nonobese phenotype in hemodialysis patients

**DOI:** 10.1186/s12902-021-00907-2

**Published:** 2021-12-19

**Authors:** Chaomin Zhou, Yanzhe Peng, Wenyong Jiang, Jing Yuan, Yan Zha

**Affiliations:** 1 Renal Division, Department of Medicine, Guizhou Provincial People’ s Hospital, Guizhou Provincial Institute of Nephritic and Urinary Disease, No. 83, Zhongshan Road, Guiyang, Guizhou China; 2 Renal Division, The First People’s Hospital of Guiyang, Guiyang, Guizhou China

**Keywords:** Visceral obesity indices, Metabolically unhealthy nonobese phenotype, Hemodialysis

## Abstract

**Background:**

Normal-weight maintenance hemodialysis (MHD) patients with abdominal obesity exhibited a more proatherogenic profile than overweight and obesity patients with abdominal obesity, highlighting the importance of early identification of metabolically unhealthy nonobese (MUNO) in this population. Visceral fat accumulation plays a crucial role in the development of MUNO. Lipid accumulation product (LAP), visceral adiposity index (VAI) have been proved as reliable visceral obesity markers. The Chinese visceral adiposity index (CVAI) and a body shape index (ABSI) are newly discovered indexes of visceral obesity and have been reported to be associated with multiple metabolic disorders. There are limited studies investigating the associations between different visceral obesity indices and risk of MUNO, especially in hemodialysis patients. Moreover, no general agreement has been reached to date regarding which of these obesity indices performs best in identifying MUNO. We aimed to investigate the prevalence of MUNO in MHD patients and compare the associations between different adiposity indices (CVAI, ABSI,VAI, LAP, body mass index (BMI), waist circumference (WC) and waist-to-hip ratio (WHtR)) with MUNO risk in this population.

**Methods:**

We conducted a multi-center cross-sectional study in Guizhou Province, Southwest China. 1302 nonobese adult MHD patients were included in our study. MUNO was defined as being nonobese and having the presence of > = 2 components of metabolic syndrome (MetS). Nonobese was defined as BMI less than 25 kg/m^2^. VAI, LAP, CVAI, ABSI, BMI, WC and WHtR were calculated. Logistic regression analyses and receiver operator curve (ROC) analyses were performed.

Results

65.6% participants were metabolically unhealthy. The ROC curve analysis demonstrated that of the seven obesity indices tested, the VAI (AUC 0.84 for women and 0.79 for men) followed by LAP (AUC 0.78 for women and 0.72 for men) had the highest diagnostic accuracy for MUNO phenotype while ABSI exhibited the lowest AUC value for identifying MUNO phenotype

**Conclusions:**

Metabolically unhealthy is highly prevalent in nonobese MHD patients. VAI and LAP outperformed CVAI in discriminating MUNO in MHD patients. Though ABSI could be a weak predictor of MUNO, it is not better than WHtR, WC and BMI.

## Background

Obesity is highly prevalent in patients with chronic kidney disease (CKD), the prevalence of obesity in CKD appears to range from 35 to 44% [1]. Several studies have demonstrated that higher body mass index (BMI) is associated with decreased mortality in patients receiving maintenance hemodialysis (MHD) therapy, known as the obesity paradox [2]. However, the survival benefits associated with higher BMI may be attenuated in participants with metabolic abnormalities [3]. Actually, BMI is not an optimal indicator in terms of assessing cardiometabolic risk associated with increased adiposity as not all obese subjects assessed by BMI show increased risk for metabolic diseases. A subgroup of obese individuals is metabolically healthy, which has been referred as “metabolically healthy obese” (MHO) phenotype [4]. Similarly, not all lean subjects could avoid the development of metabolic abnormalities. A subgroup of relatively lean individuals is at increased risk of cardiometabolic disease and is metabolically unhealthy. This condition has been defined as “metabolically unhealthy nonobese” (MUNO) phenotype [5, 6]. As is well known, Asians are predisposed to visceral or abdominal obesity and at increased risks of metabolic diseases despite with low BMI. In this regard, it becomes indispensable to stratify subjects according to metabolic status rather than obesity. A recent study suggests that normal weight MHD patients with abdominal obesity exhibit a more proatherogenic profile and lower physical ability than overweight and abdominally obese patients [7]. We speculate that the results are mainly attributed to the metabolically unhealthy characteristics of the patients. In this regard, it becomes indispensable to identify MUNO individuals in this population.

Visceral fat accumulation plays a crucial role in the development of metabolic unhealthy phenotype [8]. Visceral adiposity index (VAI) and lipid accumulation product (LAP) have been verified as reliable visceral adiposity measures by lots of studies [9, 10]. A body shape index (ABSI) and Chinese visceral adiposity index (CVAI) have been identified as two novel markers of visceral obesity recent years [11]. The new markers are reported to outperform other obesity markers in assessing all-cause mortality [12] and identifying type 2 diabetes as well as diabetic complications [13, 14]. Whether the new visceral obesity markers are associated with risk of MUNO phenotype remains unknown. Though many visceral obesity indices such as VAI, LAP and ABSI have been reported to be associated with multiple metabolic disorders, studies investigating the associations between these indices and MUNO phenotype are limited. Moreover, no general agreement has been reached to date regarding which of these indices performs best. In addition, whether the new markers perform better than the old ones as well as the traditional adiposity measures in the prediction of MUNO phenotype in MHD patients remain inconclusive.

This study aimed to investigate the prevalence of MUNO in MHD patients and further explore and compare the associations of novel visceral obesity markers and traditional obesity indices with risk of MUNO phenotype in this population.

## Methods

### Study population

A multicenter, cross-sectional study was conducted in Guizhou Province, Southwest China. Adult MHD patients who received 4-h standard twice to thrice-weekly dialysis treatment in 11 hemodialysis centers of Guizhou Province from June 1, 2017 to September 30, 2017 were recruited to our study. All these patients were clinically stable and underwent MHD for more than 3 months. A total of 2103 patients participated in our study voluntarily. We excluded participants who received HD treatment for less than 3 months. Subjects with obvious edema or ascites, hearing disabilities, physical deformities, language barriers and on any kind of nutritional support were also excluded from our study. Overweight or obese (BMI > or = 25 kg/m^2^) patients were also excluded from our final analysis. All participants provided written informed consent. This study was approved by the ethics committee of The People’s Hospital of Guizhou province.

### Data collection

As described in our previous research [15], information on age, sex, current or past cigarette smoking, educational attainment, history of current and previous illness, and medical treatment was acquired through a structured questionnaire, which is similar to that in the previous article. The questionnaires were completed by well-trained physicians through face-to-face interviews with the patients. We extracted the latest laboratory parameters from medical records. These laboratory parameters included fasting glucose, triglyceride (TG), total cholesterol (TC), high-density lipoprotein cholesterol (HDL-c), and low-density lipoprotein-cholesterol (LDL-c). Subjects who did not have a routinely blood test in the past 3 months were excluded in the final analysis.

### Measurements

All measurements were performed after a routine HD session by well-trained nurses and physicians following standardized procedures. Height and weight were obtained while the participants were barefoot and in light clothing. BMI was calculated as weight (kg) divided by the square of the height (m^2^). WC was measured with an inelastic measuring tape in centimetres at the midpoint between the last rib and the iliac crest with the patients in standing positions. WHtR was calculated as the ratio between WC and height. Blood pressure (BP) was measured on the non-fistula arm in a standardized manner by physicians in the 11 hemodialysis centers with an automatic manometer and the average of 3 readings was used in the final analysis. Pre-dialysis BP was measured before hemodialysis in a sitting position after a rest of ⩾ 5 min.

### Calculation of anthropometric indices

LAP [10], CVAI [16], VAI [10] and ABSI were calculated according to the following formulas:

LAP_men_ = (WC (cm)-65) × TG (mmol/l);

LAP_women_ = (WC (cm)-58) × TG (mmol/l);

CVAI _men_ = − 267.93 + 0.68 × age+ 0.03 × BMI (kg/m^2^) + 4.00 × WC (cm) + 22.00.

×Log10 TG (mmol/L)-16.32 × HDL-C (mmol/L);

CVAI_women_ = − 187.32 + 1.71 × age+ 4.23 × BMI (kg/m^2^) + 1.12 × WC (cm) + 39.76.

×Log10 TG (mmol/L)-11.66 × HDL-C (mmol/L);

VAI_men_ = WC∕[39.68 + (1.88 × BMI)] × TG∕1.03 × 1.31∕HDL;

VAI_women_ = WC∕[36.58 + (1.89 × BMI)] × TG∕0.81 × 1.52∕HDL;

ABSI=WC(m)/ [BMI^2/3^ (kg/m^2^) × height^1/2^(m)];

### Definition of metabolically unhealthy nonobese phenotype

Subjects are considered to have MUNO phenotype if they have a BMI that is less than 25 kg/m^2^ [9]. Furthermore, they must meet with two or more of the following abnormalities at the same time: (1) systolic BP ⩾ 130 mmHg and/or a diastolic BP ⩾ 85 mmHg, or use of antihypertensive drugs; (2) TG ⩾ 1.7 mmol/l; (3) HDL-C < 1.0 mmol/l in men and < 1.3 mmol/l in women or taking lipid-lowering drugs for the indication of hyperlipidemia; (4) fasting plasma glucose (FPG) ⩾ 5.6 mmol/l or on antidiabetic treatment for raised blood glucose [17].

### Statistical analysis

Continuous variables were presented as medians (interquartile range) due to their skewed distribution. Mann–Whitney U test was used to compare differences between metabolically healthy nonobese (MHNO) and MUNO patients. Categorical variables were expressed as frequency and proportion and differences between the two groups were analyzed by Chi-square tests. Quartiles of CVAI, VAI, LAP, ABSI, BMI, WC and WHtR were calculated and the lowest quartiles of the indexes were set as the reference. Logistic regression analyses were applied to evaluate the independent association of different adiposity indices with MUNO phenotype. The receiver operating characteristic (ROC) curves were built and areas under ROC curves (AUROCs) were calculated to estimate the diagnostic accuracy of the seven obesity indexes for MUNO phenotype. The optimal cut-off points for different adiposity indices were calculated according to the Youden indexes. Significance tests for comparison of AUCs from different obesity indices were assessed by the method described by DeLong [18]. Statistical analyses were performed with the statistical software SPSS version 16.0. Significance tests for comparison of AUCs were performed using MedCalc version 13.0 for Windows (MedCalc Software, Mariakerke, Belgium).

## Results

1302 nonobese individuals (54 ± 16 years), ranging in age from 18 to 90 years, met the inclusion criteria and were included in our final analysis. Among these individuals, 854(65.6%) participants met the criteria of MUNO phenotype and 448 subjects were metabolically healthy. The basic characteristics of the study population were listed in Table 1. Compared with metabolically healthy nonobese (MHNO) individuals, patients with the MUNO phenotype seemed to be younger and had significantly higher WC, BMI, WHtR, CVAI, VAI, LAP, FPG and TG, but lower HDL-C and diastolic BP. In addition, subjects with the MUNO phenotype exhibited a higher prevalence of history of hypertension and diabetes. No significant differences were found between the two groups for levels of LDL-C, TC, systolic BP and educational status.

**Table 1:** Baseline characteristics of the study population

Characteristics	MHNO (***n*** = 448)	MUNO (***n*** = 854)	***P*** value
Age (yr)	59 (47,70)	52 (39,65)	0.005
Male n(%)	296 (66.1%)	489 (57.3%)	0.001
History of hypertension (%)	244 (63.7%)	273 (88.9%)	< 0.001
History of diabetes (%)	446 (13.0%)	596 (35.8%)	< 0.001
History of smoking (%)	214 (41.5%)	146 (35.6%)	0.04
Educational status (≥junior high school)(%)	34.8%	36.3%	0.35
Systolic BP (mmHg)	140 (125,154)	140 (127,154)	0.9
Diastolic BP (mmHg)	80.0 (70,89.5)	77 (68,88)	0.003
WC (cm)	77.5 (72,84.2)	82 (75.5,87)	< 0.001
BMI (kg/m^2^)	20.35 (18.91,22.20)	21.46 (19.63,23.06)	< 0.001
WHtR	0.48 (0.45,0.53)	0.51 (0.47,0.54)	< 0.001
CVAI	59.97 (31.24,91.72)	87.47 (57.04,112.64)	< 0.001
VAI	1.17 (0.82,1.59)	2.40 (1.43,3.73)	< 0.001
LAP	15.19 (8.58,23.94)	29.50 (16.37,51.94)	< 0.001
ABSI	0.082 (0.078,0.087)	0.084 (0.080,0.088)	< 0.001
FPG (mmol/L)	4.73 (4.35,5.17)	6.22 (4.99,8.10)	< 0.001
TG (mmol/L)	1.05 (0.81,1.33)	1.62 (1.1,2.31)	< 0.001
HDL-C (mmol/L)	1.31 (1.12,1.55)	1.02 (0.84,1.26)	< 0.001
LDL-C (mmol/L)	2.10 (1.64,2.75)	2.20 (1.70,2.78)	0.24
TC (mmol/L)	3.87 (3.24,4.42)	3.89 (3.31,4.60)	0.18

Abbreviations: *WC* waist circumference, *BMI* body mass index, *WHtR* waist-height ratio, *CVAI* Chinese visceral adiposity index, *VAI* visceral adiposity index, *LAP* lipid accumulation product, *ABSI* a body shape index, *BP* Blood pressure, *FBG* fasting blood-glucose, *TG* triglyceride, *HDL-c* high-density lipoprotein cholesterol, *LDL-c* low-density lipoprotein-cholesterol, *TC* total cholesterol

### Risk of MUNO phenotype by quartiles of different adiposity indices

As shown in Table 2, all adiposity indices included in our study were independently associated with MUNO phenotype risk, even after adjustment for potential intermediate variables such as age, sex, educational status and history of smoking. The adjusted ORs for MUNO phenotype rose progressively with increasing quartiles of adiposity indices except for ABSI.

**Table 2:** Odds ratio (95% CI) of the presence of MUNO for different adiposity indices after adjustment of age, sex, educational status and history of smoking

Obesity indexes	Quartile 1	Quartile 2	Quartile 3	Quartile 4
BMI	1	1.01 (0.70–1.45)	2.07 (1.41–3.05)^※^	2.20 (1.48–3.28)^※^
WC	1	1.53 (1.05–2.22)^*^	2.73 (1.82–4.07)^※^	2.96 (1.94–4.51)^※^
WHtR	1	1.74 (1.19–2.54)^*^	2.49 (1.69–3.66)^※^	3.11 (2.00–4.85)^※^
VAI	1	2.35 (1.60–3.44)^※^	10.01 (6.38–15.71) ^※^	88.18 (34.56–225.02) ^※^
CVAI	1	3.39 (2.19–5.25)^※^	8.08 (4.82–13.55)^※^	13.23 (7.39–23.68)^※^
ABSI	1	1.75 (1.21–2.55)^*^	2.08 (1.42–3.06)^※^	1.97 (1.33–2.92)^※^
LAP	1	1.76 (1.82–4.69)^*^	3.51 (2.35–5.22)^※^	30.16 (15.81–57.55)^※^

The between cut points are 19.27, 21.17, 22.87 for BMI; 74.50, 80.35, 86.23for WC; 0.46, 0.50, 0.54 for WHtR; 1.14, 1.74, 3.09 for VAI; 48.4, 79.33, 107.4 for CVAI; 0.079, 0.083, 0.088 for ABSI; 12.80, 23.19, 39.68 for LAP; *P < 0.05, ※ < 0.001

Abbreviations: *BMI* body mass index, *WC* waist circumference, *WHtR* waist-to-height ratio, *VAI* visceral adiposity index, *CVAI* Chinese visceral adiposity index, *ABSI* a body shape index, *LAP* lipid accumulation product

### ROC analyses for different adiposity indices to predict the risk of MUNO phenotype

**Fig. 1:**
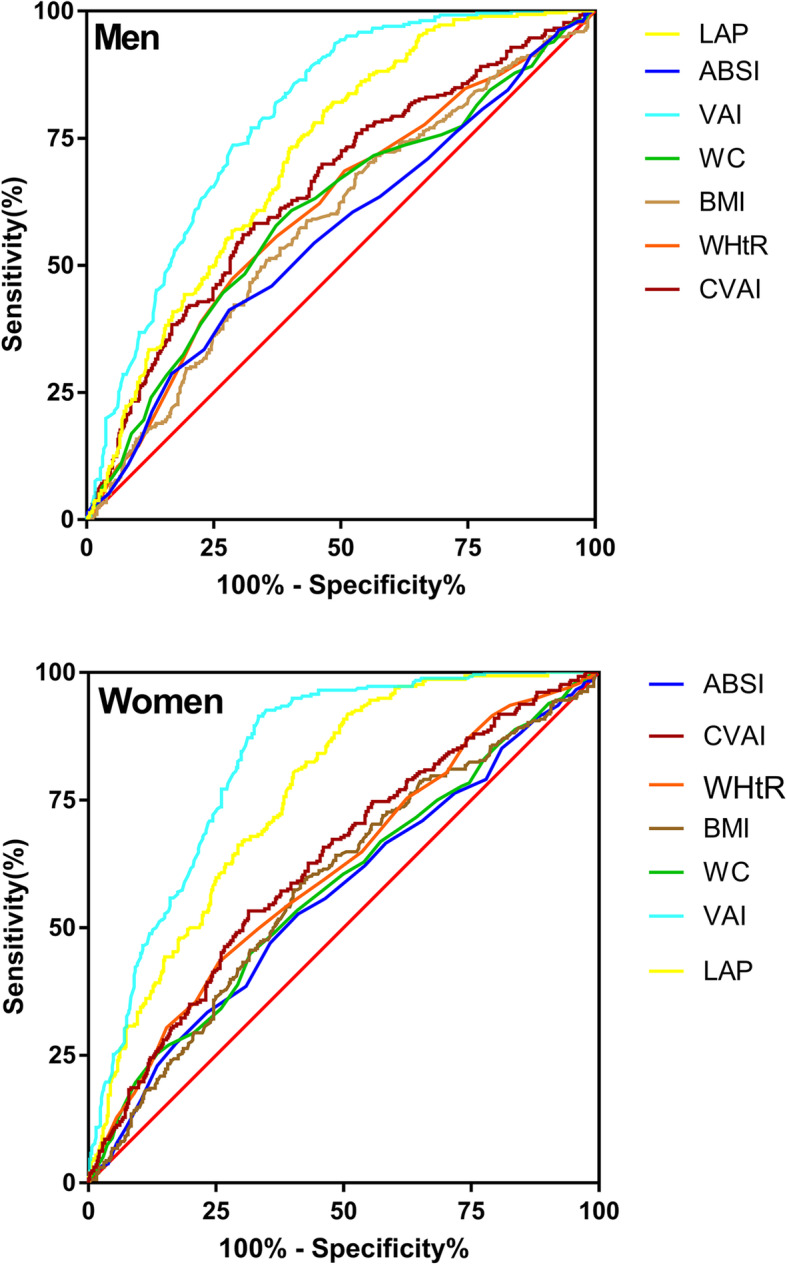
ROC curves of the seven obesity indices to diagnose MUNO phenotype in men and women. Legend: BMI, body mass index; WC, waist circumference; WHtR, waist-to-height ratio;VAI, visceral adiposity index; CVAI, Chinese visceral adiposity index; ABSI, a body shape index; LAP, lipid accumulation product. ROC, receiver operating characteristic

As shown in Table 3 and Fig. 1, all the obesity indices included in the study were able to discriminate MUNO phenotype (all area under the ROC curves [AUCs] > 0.5, *P* < 0.05). The ROC curve analysis demonstrated that of the seven obesity indices tested, the VAI (AUC 0.84 for women and 0.79 for men) followed by LAP (AUC 0.78 for women and 0.72 for men) had the highest diagnostic accuracy for MUNO phenotype while ABSI was the least accurate predictor of MUNO phenotype for both men and women. The best VAI value for diagnosis of MUNO phenotype was 1.71(sensitivity 0.60, specificity 0.86) for men and 2.0 (sensitivity 0.72, specificity 0.87) for women.

**Table 3:** Comparison of diagnostic performance of novel visceral obesity indices with old ones and traditional adiposity indices

Adiposity indices	AUC (95% CI)	p	Cut-off	Sensitivity	Specificity	Youden Index
**Males**						
BMI	0.58 (0.55–0.62)	< 0.001	20.69	0.65	0.51	0.16
WC	0.60 (0.57–0.64)	< 0.001	81.5	0.59	0.62	0.21
WHtR	0.61 (0.57–0.64)	< 0.001	0.48	0.68	0.53	0.20
VAI	0.79 (0.76–0.82)	< 0.001	1.71	0.60	0.86	0.46
CVAI	0.65 (0.62–0.69)	< 0.001	72.39	0.67	0.58	0.25
ABSI	0.56 (0.53–0.60)	0.005	0.080	0.73	0.41	0.14
LAP	0.72 (0.68–0.75)	< 0.001	24.38	0.53	0.80	0.34
**Females**						
BMI	0.64 (0.60–0.69)	< 0.001	20.96	0.56	0.72	0.27
WC	0.64 (0.60–0.68)	< 0.001	72.8	0.82	0.41	0.23
WHtR	0.62 (0.58–0.66)	< 0.001	0.47	0.83	0.38	0.21
VAI	0.84 (0.80–0.87)	< 0.001	2.00	0.72	0.87	0.59
CVAI	0.70 (0.66–0.74)	< 0.001	72.22	0.61	0.71	0.33
ABSI	0.56 (0.52–0.61)	0.03	0.084	0.53	0.61	0.14
LAP	0.78 (0.73–0.81)	< 0.001	30.08	0.60	0.84	0.43

Abbreviations: *BMI* body mass index, *WC* waist circumference, *WHtR* waist-to-height ratio, *VAI* visceral adiposity index; *CVAI* Chinese visceral adiposity index, *ABSI* a body Abbreviations shape index, *LAP* lipid accumulation product

### Significance tests of AUCs from different obesity indices for prediction of MUNO phenotype

Differences between the AUCs of VAI with LAP; WHtR with CVAI and ABSI; WC with WHtR, ABSI and CVAI; BMI with WC, WHtR and ABSI were further tested as their 95% CI were overlapped. BMI showed similar discriminative power with WC, WHtR and ABSI in both genders based on non-significant differences in AUC tests (shown in Table 4). WC performed better than WHtR in predicting MUNO phenotype in women(*P* = 0.02), while WC showed similar discriminative power with WHtR in men(*P* = 0.87). CVAI showed higher AUC than WC and WHtR for detection of MUNO in both men and women. VAI outperformed LAP showed lower AUC (in comparison with VAI), whereas WC and WHtR showed better predictive capacity of MUNO than ABSI(*P* < 0.05).

**Table 4:** Significance tests for comparison of AUCs from different obesity indices

Characteristics	Male		Female	
	*Z*	*P*	*Z*	*P*
BMI vs WC	0.993	0.32	0.457	0.65
BMI vs WHtR	1.111	0.27	0.377	0.71
BMI vs ABSI	1.012	0.31	1.307	0.19
WC vs WHtR	0.158	0.87	2.276	0.02
WC vs ABSI	2.776	0.006	3.260	0.0001
WC vs CVAI	6.223	< 0.0001	2.116	< 0.0001
WHtR vs CVAI	4.481	< 0.0001	3.054	0.03
WHtR vs ABSI	2.776	0.006	2.013	0.035
LAP vs VAI	4.487	< 0.0001	3.306	< 0.0001

Abbreviations: *BMI* body mass index, *WC* waist circumference, *WHtR* waist-to-height ratio, *VAI* visceral adiposity index; *CVAI* Chinese visceral adiposity index, *ABSI* a body Abbreviations shape index, *LAP* lipid accumulation product

## Discussion

Our study demonstrated that 65.6% participants were metabolically unhealthy, despite with relatively low BMI. All the seven adiposity indices included in our study were independently associated with increased risk for MUNO phenotype. VAI and LAP showed significantly higher AUCs than CVAI while ABSI was inferior to traditional anthropometric indices in discriminating MUNO phenotype.

A more proatherogenic profile in MHD patients with normal weight abdominal obesity than obese or overweight MHD patients highlighted the importance of exploring the relationship between visceral/abdominal obesity and metabolically unhealthy in nonobese MHD patients [7]. Previous studies have suggested that visceral obesity contributes a lot to the development of MUNO phenotype [19]. Searching for simple and effective surrogate markers of visceral obesity has acquired more and more attentions since direct estimation of visceral fat demands imaging diagnostic tests which are costly and have low availability for epidemiological use. VAI and LAP are two reliable visceral obesity indices developed and validated in Caucasian population. But differences in body fat distribution should be considered among different ethnicity. It is widely known Chinese are more prone to be visceral obese despite having relatively low BMI. CVAI, a new visceral obesity index developed and validated in Chinese population, was found to be superior to other obesity markers in identifying cardiometabolic risk factors [16, 20–22]. However, CVAI is inferior to old visceral obesity indices such as VAI and LAP in discriminating MUNO phenotype in our study. In fact, the superiority of VAI in assessing metabolic risk has been reported by lots of studies [17, 23]. Our finding that VAI followed by LAP, CVAI presented the highest AUROC for MUNO phenotype highlighted the supremacy of VAI in predicting MUNO phenotype. Similar to the general population, increasing BMI demonstrated a positive association with the MUNO phenotype in our study. This was somewhat consistent with a previous study which demonstrated that normal-weight patients on hemodialysis with abdominal obesity exhibited a more proatherogenic profile than abdominal obesity patients with overweight and obesity [7]. Participants with higher BMI might be abdominal obese as most of the participants were middle-aged (mean age, 54 ± 16 years), and as we all known, middle-aged people were prone to abdominal obesity. Consistent with our study, a previous study also suggested that VAI followed by LAP was a better indicator of MUNO phenotype compared to BMI, WC and WHtR [10].

Exact mechanisms accounting for the different predictive value of CVAI in our study and previous studies are not known. Fat distribution may vary in different diseases and the characteristics of the study population are different. Participants with MUNO phenotype were relatively younger than those with MHNO phenotype in our study. A previous study has suggested that visceral adipose tissue (VAT) was not associated with incident atherosclerotic cardiovascular disease events in older men [24]. We speculated that VAT might play a protective role in metabolic risk in relatively older MHD patients. CVAI is calculated based on age, BMI, WC, TG, and HDL-C, but the establishment of VAI is not affected by age. Therefore, VAI outperformed CVAI as it is not affected by age in identifying metabolically unhealthy obesity in our study. Furthermore, though CVAI has been proposed to be a reliable Chinese-based marker of visceral obesity, only a small group of the subjects in the study of Xia et al. received abdominal computed tomography (CT), which served as a gold method for visceral fat assessment [16]. Furthermore, AUROCs of WC and BMI for visceral fat evaluation in their study were even higher than that of VAI, which implied WC and BMI were better predictors of visceral obesity than VAI. This is obviously inconsistent with previous researches. More studies that directly measure visceral fat areas in Chinese population are needed to validate the predictive value of CVAI in evaluating visceral obesity.

ABSI, another new marker of visceral obesity, was reported to be a stronger predictor of diabetes than BMI [25] and was superior to traditional measures of adiposity in assessing carotid atherosclerosis [26]. In addition, ABSI achieves better mortality risk stratification than abdominal obesity indices [12]. However, the AUROC of ABSI for diagnosis of MUNO phenotype in our study was the lowest. Comparison of AUCs showed that WC and WHtR performed better than ABSI in discriminating MUNO while BMI share similar AUC with ABSI. In accordance with our results, a previous study suggested that VAI was superior to ABSI, WC, BMI and other anthropometric indices in assessing the risk of metabolic syndrome (MetS) [27]. As we all known, patients with MetS share similar metabolic profiles to those with MUNO phenotype. Our results were further strengthened by a previous study demonstrating that ABSI was a weaker predictor of MetS than WC and BMI [28]. Exact mechanisms accounting for the disadvantage of ABSI in predicting MUNO phenotype in our study are unknown. It might be attribute to the fact that ABSI doesn’t take TG into account, but increased TG is associated with insulin resistance even in subjects with normal BMI and normal glucose tolerance [29] and insulin resistance is the core characteristic of MUNO phenotype. Furthermore, many studies have suggested that indexes composed of TG are very suitable for the identification of metabolically unhealthy individuals [30].

Several limitations of our study should be considered when interpreting the findings. Firstly, the cross-sectional nature of the study disabled us to make causality between the obesity indices and MUNO risk. Secondly, high blood pressure is a key factor associates with the definition of MUNO phenotype. Most dialysis patients in our study had hypertension and this might attribute to the kidney diseases but not metabolic abnormality. Thus, the prevalence of MUNO might be exaggerated. Further studies are needed to clarify whether the definition of MUNO phenotype is suitable for dialysis patients. Thirdly, since mean age of our patients were 54 ± 16 years, our results and conclusions might be more suitable for middle-aged and elderly Chinese MHD patients. Fifth, some of the components included in the investigated indices are identical to the components included in the MUNO criteria. The mathematical coupling can inflate regression coefficients and AUCs, so the results should be interpreted with caution. Extrapolating our findings to other populations should be interpreted cautiously.

## Conclusions

Metabolically unhealthy is highly prevalent in nonobese MHD patients. VAI and LAP outperformed CVAI in discriminating MUNO phenotype in MHD patients. Though ABSI could be a weak predictor of MUNO phenotype, it is not better than WHtR, WC and BMI.

## Data Availability

The datasets used and/or analysed during the current study are available from the corresponding author on reasonable request.

## Abbreviations

BMI: Body mass index
WC: Waist circumference
WHtR: Waist-to-height ratio
VA: Visceral adiposity index
CVAI: Chinese visceral adiposity index
ABSI: A body abbreviations shape index
LAP: Lipid accumulation product
MHD: Maintenance hemodialysis
MUNO: Metabolically unhealthy nonobese
MetS: Metabolic syndrome
TG: Triglyceride
TC: Total cholesterol
HDL-c: High-density lipoprotein cholesterol
LDL-c: Low-density lipoprotein-cholesterol
ROC: Receiver operating characteristic
